# Differences in the determinants of right ventricular and regional left ventricular long-axis dysfunction in Friedreich ataxia

**DOI:** 10.1371/journal.pone.0209410

**Published:** 2018-12-31

**Authors:** Roger E. Peverill, Lesley Donelan, Louise A. Corben, Martin B. Delatycki

**Affiliations:** 1 Monash Cardiovascular Research Centre, MonashHeart and Department of Medicine (School of Clinical Sciences at Monash Health), Monash University and Monash Health, Clayton, Victoria, Australia; 2 Bruce Lefroy Centre for Genetic Health Research, Murdoch Children’s Research Institute, Parkville, Victoria, Australia; 3 Victorian Clinical Genetics Services, Parkville, Victoria, Australia; Indiana University, UNITED STATES

## Abstract

**Background:**

Friedreich ataxia (FRDA) is an autosomal recessive neurodegenerative condition which also has effects on the heart. In 96% of affected individuals FRDA is due to homozygosity of a GAA repeat expansion in intron 1 of the frataxin (*FXN)* gene. The number of GAA repeats have been shown to relate to disease severity in FRDA, this thought to be via an inverse relationship of GAA repeat number and cellular frataxin levels. We investigated the effects of FRDA on regional long axis function of the left and right ventricles, and also the relationship of long axis systolic (s`) and early diastolic (e`) peak velocities with GAA repeat number on the shorter (GAA1) and longer *FXN* alleles (GAA2).

**Methods:**

The study group of 78 adult subjects (age 32±9 years) with FRDA and normal left ventricular (LV) ejection fraction were compared to 54 healthy control subjects of similar age, sex and body size. Tissue Doppler imaging (TDI) signals were recorded at the mitral annulus for measurement of s`and e`of the septal, lateral, anterior and inferior walls and at the tricuspid annulus for measurement of right ventricular (RV) s`and e`.

**Results:**

All the regional LV s`and e`, and both RV s`and RV e`, were lower in individuals with FRDA compared to controls (p<0.001 for all). On multivariate analysis, which included LV septal wall thickness (SWT), RV s`and RV e`were both inversely correlated with GAA1 (β = -0.32 & -0.33, respectively, p = 0.01), but not with GAA2, whereas anterior and lateral s`were both inversely correlated with GAA2 (β = -0.25 and β = -0.28, p = 0.02) but not with GAA1. Increasing SWT was the most consistent LV structural correlate of lower s`and e`, whereas age was a consistent inverse correlate of e`but not of s`.

**Conclusion:**

There are generalized abnormalities of both LV regional and RV long axis function in FRDA, but there are also regional differences in the association of this dysfunction with the smaller and larger GAA repeats in the *FXN* gene.

## Introduction

Friedreich ataxia (FRDA) is an autosomal recessive neurodegenerative condition due to a deficiency in the mitochondrial protein frataxin (*FXN*), and in 96% of affected individuals it is due to homozygosity of a GAA repeat expansion in intron 1 of the *FXN* gene [1]. Cardiac disease is a frequent accompaniment of FRDA and can lead to arrhythmias, cardiac failure and premature death [2,3]. Left ventricular (LV) structural involvement prior to both cardiac symptom onset and reduction in LV ejection fraction in FRDA has been documented in a number of studies, with increases in LV wall thickness, reduction in LV chamber size and therefore an increase in LV relative wall thickness (RWT), being commonly reported findings [4,5]. LV function can also be impaired in FRDA prior to cardiac symptom onset and with a LV ejection fraction within the normal range, and several studies have reported abnormalities of long axis LV function in FRDA in subjects with preserved LV ejection fraction [4,6,7].

There is evidence that there may be early regional differences in the progression of LV dysfunction in FRDA, with the posterior wall being adversely affected preferentially in one study [8] and differences between the septal and lateral LV walls evident in a second study [4]. One possible contribution to regional differences in LV myocardial function is that the septum is shared by both left and right ventricles and thus may behave differently from other LV walls [9]. However, the effects of FRDA on regional LV long-axis function and the relationship of GAA repeats with regional LV long-axis function have not been examined in detail, whereas the limited available data suggests that the right ventricular (RV) long-axis function may not be affected in FRDA [6].

To further define the effects of FRDA on myocardial function, we investigated the effects of FRDA on long axis systolic and early diastolic tissue Doppler imaging (TDI) velocities of the RV free wall, and of the septal, inferior, anterior and lateral walls of the left ventricle. In addition, we investigated the relationship of the RV and LV regional velocities with GAA expansion length of both the smaller (GAA1) and larger *FXN* alleles (GAA2). The study group comprised adult subjects with FRDA due to homozygosity for GAA expansions in the *FXN* gene who had a normal LV ejection fraction (>50%) and who were free of hypertension and diabetes. Subjects with FRDA were compared to a control group of similar age, sex and blood pressure.

## Methods

### Subjects

All research involving human participants was approved by the Monash Health Human Research and Ethics Committee and all clinical investigation was conducted according to the principles expressed in the Declaration of Helsinki. The echocardiographic studies of individuals with FRDA who met the study criteria were identified retrospectively, the need for consent was waived, and the analysis was performed on anonymized data. Written informed consent was obtained from all control subjects. The study group comprised 78 adult subjects with FRDA due to homozygosity for a GAA triplet repeat expansions in intron 1 of the *FXN* gene who were seen in a multidisciplinary FRDA clinic and who did not meet the exclusion criteria. FRDA subjects were excluded if they had diabetes, a history of heart failure or hypertension, a cardiac rhythm other than sinus, a blood pressure (BP) greater than 140/90 mmHg at the time of the echocardiographic examination, an ejection fraction ≤50% or more than mild valvular disease. Data from the same echocardiogram for M-mode LV measurements and septal and lateral TDI velocities has been reported previously for 27 of the subjects in the present study [4]. The individuals with FRDA were compared to 54 healthy control subjects with normal blood pressure, no known cardiac disease, and of similar sex proportion and age to the FRDA group.

### Echocardiography

Transthoracic echocardiography was performed using a Sonos 5500 ultrasound machine (Philips) and measurements were performed off-line using Xcelera V1.2 L4 SP2 (Philips). M-mode images of the left ventricle were obtained in the parasternal long axis view just distal to the mitral valve leaflet tips after alignment of the cursor perpendicular to the left ventricular wall. 2-D images were also used to facilitate identification of the endocardium and standard M-mode measurements of LV septal wall thickness (SWT), posterior wall thickness (PWT), LV end-diastolic diameter (LVEDD) and LV end-systolic diameter were obtained [10,11]. Fractional shortening (FS) was calculated by the standard formula. LV mass (LVM) was calculated using the modified formula of Devereux et al [12] and divided by body surface area (BSA) to calculate LV mass index (LVMI). Relative wall thickness (RWT) was calculated as 2 times the PWT divided by the LV end-diastolic diameter.

Four- and two-chamber apical loops of left ventricular contraction were recorded and used for measurement of LV EF using the biplane method of discs. The length of the LV at end-diastole from the plane of the mitral annulus to the apical endocardium in the 4- and 2-chamber views was recorded during the end-diastolic measurement, and the longest dimension from these 2 views was used as the left ventricular end-diastolic length (LVEDL) [13,14].

### Tissue Doppler imaging

Pulsed wave TDI was performed in the apical four- and two-chamber apical views. TDI velocities of longitudinal annular motion were recorded at end expiration apnoea at the septal, lateral, anterior and inferior mitral annular borders and at the lateral tricuspid annular border, all after optimizing parallel alignment of the ultrasound beam. Spectral pulsed wave Doppler was used with instrument settings adjusted to record the high frequency/low velocity tissue signals using an appropriate sample volume size. Peak systolic (s`), and early diastolic (e`) velocities were measured off-line and 3 consecutive beats were averaged. Satisfactory tissue Doppler signals were acquired in all subjects. Interobserver variability for measurements of TDI velocities in control subjects in our laboratory using the Sonos 5500 have been reported previously and varied between 5.5 and 7.7% [15].

### Analysis of GAA expansion

The number of GAA repeats in the *FXN* gene was measured as previously described [16].

### Statistics

Data were analyzed using standard statistical software (SYSTAT V13). Continuous variables are presented as mean ± SD. Group differences in continuous variables between FRDA subjects and controls were assessed with an unpaired Student t test. Linear regression analysis was performed in FRDA subjects to determine correlations of RV and LV regional TDI velocities with independent variables including age, age at the onset of symptoms (AOS), symptom duration, GAA1, GAA2, heart rate, blood pressure, body surface area (BSA), body mass index (BMI), LVEDD, SWT and LVEDL [15,17–19].

The correlation coefficient (r) is provided for univariate regression analyses and the partial correlation coefficient (β is provided in those multivariate regression analyses where an aim was to determine the degree of contribution of various independent variables to the dependent variable. The adjusted value of the coefficient of determination (adjusted r^2^) has been provided for multivariate analyses as an estimate of the degree of variance in a dependent variable explained by a multivariate model after adjustment for the number of independent variables in the model. Apart from decisions regarding inclusion of variables in multivariate models, a p value of <0.05 was considered significant.

## Results

The baseline characteristics of the FRDA and control group and details of GAA1, GAA2, AOS and symptom duration in the FRDA group are shown in Table 1. FRDA subjects had a similar age, BSA, BMI and BP to the control group, but heart rate was higher in the FRDA group. The standard echocardiographic measurements of LV size and function are shown in Table 2. FRDA subjects had increased SWT, PWT and RWT but a reduced LVEDD and LVEDL. In FRDA subjects, all LV measurements other than RWT and FS were larger in males than females (p<0.05 for all) and this included LVMI and also LVEDD indexed to BSA (p<0.05 for both). LVMI was greater in FRDA than control subjects in both males and females. The ejection fraction was similar in the FRDA and control groups but FS was higher in the FRDA group.

**Table 1:** Demographic, anthropometric and disease specific characteristics in individuals with Friedreich ataxia and control subjects.

	FRDA n = 78	Controls n = 54	p
Male / Female	35/43	25/29	NS
Age (years)	31.1±9.3	31.9±8.8	NS
Height (cm)	170±10	169±8	NS
Weight (kg)	68.5±13.5	67.5±11.7	NS
BSA (m^2^)	1.79±0.20	1.77±0.18	NS
BMI (kg/m^2^)	23.6±4.4	23.5±2.9	NS
Heart rate (/min)	74.6±10.8	62.4±10.6	<0.001
Systolic BP (mmHg)	111±14	116±12	NS
Diastolic BP (mmHg)	69±10	68±10	NS
GAA1	662±195		
GAA2	881±205		
Age at onset of symptoms (years)	13.5±4.9		
Symptom duration (years)	18.1±9.5		

BMI—body mass index, BP—blood pressure, BSA—body surface area, GAA1—number of GAA repeats in the shorter allele of *FXN*, GAA2—number of GAA repeats in longer allele of *FXN*

**Table 2:** Left ventricular structure and function in individuals with Friedreich ataxia and control subjects.

	FRDA	Controls	P
LVEDD (cm)	4.6±0.5	4.8±0.4	0.001
SWT (cm)	1.1±0.2	0.8±0.1	<0.001
PWT (cm)	1.1±0.2	0.8±0.1	<0.001
RWT	0.49±0.12	0.34±0.05	<0.001
LVEDL (cm)	8.1±0.7	8.7±0.7	<0.001
LV mass index (g/m^2^)			
Male	115±34	87±17	<0.001
Female	88±19	67±16	<0.001
Fractional shortening (%)	40.3±7.4	36.2±5.3	<0.001
Ejection fraction (%)	65±7	65±6	0.70

LV—left ventricular, LVEDD, left ventricular end-diastolic diameter, LVEDL—left ventricular end-diastolic length, PWT—posterior wall thickness, RWT—relative wall thickness, SWT—septal wall thickness

On multivariate analysis in FRDA subjects, LVEDL was larger in males (p<0.001), positively correlated with BSA (p = 0.033) and inversely correlated with age (p = 0.042) and GAA1 (p = 0.028). LVEDD was also larger in males (p = 0.003) and positively correlated with BSA (p = 0.024), but it was not correlated with age and it was only borderline correlated with GAA1 (p = 0.074). LVM was larger in males (p<0.001), positively correlated with BSA (p = 0.038) and inversely correlated with age (p = 0.004), but was not related to GAA1. SWT was larger in males (p = 0.006) and inversely correlated with age (p = 0.034), but there was no correlation of SWT with BSA or GAA1.

### Comparison of tissue Doppler measurements with control subjects

Comparison of s`and e`at the tricuspid annulus and the septal, inferior, anterior and lateral borders of the mitral annulus in FRDA and control subjects are shown in Table 3. RV s`and e`and all the LV s`and e`velocities were lower in FRDA subjects by comparison to the control group. The normal pattern of regional LV TDI s`and e`velocities was evident in both the control and FRDA groups, this being lowest velocities at the septal wall and highest velocities at the lateral wall [20].

**Table 3:** Comparison of right ventricular and left ventricular regional long axis systolic and early diastolic velocities in individuals with Friedreich ataxia and control subjects.

	FRDA	Controls	P
RV s`(cm/s)	11.6±2.3	13.8±2.4	<0.001
Septal s`(cm/s)	7.3±1.3	8.4±1.5	<0.001
Inferior s`(cm/s)	7.9±1.7	9.5±1.6	<0.001
Anterior s`(cm/s)	9.0±2.6	10.3±1.8	0.001
Lateral s`(cm/s)	9.5±2.7	11.3±2.2	<0.001
RV e`(cm/s)	11.0±2.9	12.9±2.6	<0.001
Septal e`(cm/s)	8.3±2.0	10.7±2.4	<0.001
Inferior e`(cm/s)	9.4±2.8	13.5±3.6	<0.001
Anterior e`(cm/s)	11.0±3.1	15.1±3.2	<0.001
Lateral e`(cm/s)	11.8±3.4	17.3±4.3	<0.001

### Correlations between RV and LV regional s`and e`in FRDA subjects

For correlations of RV s`with LV regional s`velocities, the highest r values were for the septal and inferior walls (r = 0.49–0.52, p<0.001 for both) and the lowest were for the anterior and lateral walls (r = 0.33–0.36, p<0.001 for both). The highest r value for RV e`was with septal e`(r = 0.64, p<0.001), and there were only small differences with the correlations of RV e`with the other LV walls (r = 0.53–0.56, p<0.001 for all). For inter-relationships of LV regional s`, the r values were highest between the septal and inferior walls, and between the anterior and lateral walls (r = 0.78 and r = 0.79, respectively, P<0.001 for both). There were similar correlations for e`between the septal and inferior walls and the anterior and lateral walls (r = 0.76 and r = 0.79, respectively, p<0.001 for both).

### Potential confounding relationships in FRDA subjects

Prior to multivariate analyses of TDI velocities in FRDA subjects, the possibility of confounding relationships were examined between the potential independent variables. There was the expected strong correlation between age and symptom duration (r = 0.84, p<0.001) and inverse correlation between GAA1 and AOS (r = -0.38, p = 0.001), but no relation between GAA2 and AOS (r = -0.07), despite there being a positive correlation between GAA1 and GAA2 (r = 0.38, p = 0.001). There were no significant correlations of GAA1 or GAA2 with either age or symptom duration. There was an inverse correlation between GAA1 and systolic BP (r = -0.23, p = 0.048), but no significant correlation of GAA1 with diastolic BP, or of GAA2 with systolic or diastolic BP. There were no correlations of LVEDD with either SWT or PWT.

### Determinants of s`

In univariate analyses of control subjects, BSA was a positive correlate of all the RV and LV s`velocities (p<0.05 for all), whereas age, heart rate, LVEDD and SWT were not correlates of any of the s`velocities. The univariate correlates of RV and LV s`velocities in FRDA subjects with age, GAA1, GAA2, AOS, symptom duration, BSA, heart rate, SWT, LVEDD and LVEDL are shown in Table 4. None of the systolic velocities were correlated with age, BSA or heart rate. There were differences between the right and left ventricles and some patterns of wall involvement evident for regional LV wall s`correlations. RV s`was inversely correlated with GAA1, but not with GAA2, whereas anterior and lateral s`were both inversely correlated with GAA2 and there was a borderline inverse correlation of anterior s`with GAA1. In contrast, septal and inferior s`were not correlated with either GAA1 or GAA2. RV, septal and inferior s`, but none of the other systolic velocities, were positively correlated with AOS, whereas only lateral s`was inversely correlated with symptom duration. SWT was inversely correlated with RV s`and all the LV s`except for the septal wall. LVEDD was positively correlated with inferior and lateral s`and LVEDL was positively correlated with anterior and lateral s`, but neither LVEDD nor LVEDL were correlated with septal s`.

**Table 4:** Correlations of right ventricular and left ventricular regional systolic long axis velocities with FRDA-specific and cardiac characteristics in FRDA subjects.

		RV s`	Septal s`	Inferior s`	Anterior s`	Lateral s`
Age	r	-0.03	0.14	0.10	-0.05	-0.13
	p	NS	NS	NS	NS	NS
GAA1	r	-0.31	-0.15	-0.24	-0.25	-0.19
	p	<0.005	NS	0.038	0.024	NS
GAA2	r	-0.24	-0.07	-0.11	-0.33	-0.30
	p	0.039	NS	NS	0.004	0.008
AOS	r	0.28	0.38	0.45	0.21	0.18
	p	0.005	0.002	<0.001	NS	NS
Symptom duration	r	-0.17	-0.06	-0.15	-0.16	-0.24
	p	NS	NS	NS	NS	0.045
BSA	r	-0.15	0.09	0.13	0.10	0.01
	p	NS	NS	NS	NS	NS
Heart rate	r	-0.06	-0.03	0.09	0.05	-0.04
	p	NS	NS	NS	NS	NS
SWT	r	-0.26	-0.17	-0.31	-0.30	-0.26
	p	0.033	NS	0.006	0.007	0.026
LVEDD	r	0.19	0.15	0.21	0.17	0.23
	p	NS	NS	NS	NS	NS
LVEDL	r	0.02	0.17	0.19	0.25	0.28
	p	NS	NS	NS	0.031	0.016

See Tables 1 and 2 for abbreviations

On multivariate modeling of RV s`and regional LV s`velocities in FRDA subjects, GAA1, GAA2, AOS, SWT, LVEDD and LVEDL were added stepwise and in that sequence, only significant correlates remained in the model for the next step, and the significant independent correlates are shown in Table 5. GAA1 was only an independent predictor of RV s`, whereas GAA2 was an independent predictor of both anterior and lateral s`. AOS was an independent positive correlate of both septal and inferior s`. SWT was an independent inverse correlate of inferior, anterior and lateral s`and LVEDL was an independent and positive correlate of inferior, anterior and lateral s`. LVEDD was not a contributor to models of LV s`once SWT was included in the model and is thus not shown in the Table.

**Table 5:** Multivariate models of RV s`and LV regional s`in adult subjects with FRDA.

		RV s`	Septal s`	Inferior s`	Anterior s`	Lateral s`
GAA1	β	-0.31				
	p	0.005				
GAA2	β				-0.28	-0.25
	p				0.006	0.005
AOS	β		0.35	0.39		
	p		0.001	<0.001		
SWT	β			-0.30	-0.36	-0.31
	p			0.003	0.001	0.04
LVEDL	β			0.24	0.30	0.32
	p			0.021	0.005	0.003
	Adjusted r2	0.09	0.11	0.28	0.24	0.21

AOS—age at onset of symptoms, For other abbreviations see Tables 1 and 2

### Determinants of e`

On univariate analysis in control subjects, all the regional LV e`were inversely correlated with age (p<0.004 for all), whereas an inverse correlation of RV e`with age was borderline significant (p = 0.086). All LV e`velocities except inferior e`were also inversely correlated with diastolic BP (0<0.04) in control subjects, whereas septal e`, but none of the other e`velocities, was inversely correlated with BMI (p<0.04). None of the control subject regional e`velocities were correlated with SWT, LVEDD or LVEDL. The univariate correlations in FRDA subjects of the various RV and LV e`velocities with age, GAA1, GAA2, AOS, symptom duration, diastolic BP, BMI, SWT, LVEDD and LVEDL are shown in Table 6. RV e`and all the LV e`velocities were inversely correlated with age. RV e`was inversely correlated with both GAA1 and GAA2 and septal e`was inversely correlated with GAA2, but none of the other LV e`velocities were correlated with either GAA1 or GAA2. All the TDI velocities were inversely correlated with symptom duration, but none were correlated with AOS, diastolic BP or BMI. All of the e`velocities were inversely correlated with SWT, all of the LV e`velocities were positively correlated with LVEDD, whereas LVEDL was not correlated with any of the velocities.

**Table 6:** Correlations of right ventricular and left ventricular regional early diastolic long axis velocities with disease- and non-disease-specific characteristics in individuals with Friedreich ataxia.

		RV e`	Septal e`	Inferior e`	Anterior e`	Lateral e`
Age	r	-0.24	-0.27	-0.27	-0.23	-0.41
	p	0.036	0.018	0.016	0.047	<0.001
GAA1	r	-0.36	-0.11	-0.06	-0.21	-0.13
	p	0.001	NS	NS	NS	NS
GAA2	r	-0.32	-0.25	-0.14	-0.19	-0.15
	p	0.004	0.028	NS	NS	NS
AOS	r	0.12	0.10	0.21	0.18	0.16
	p	NS	NS	NS	NS	NS
Symptom duration	r	-0.31	-0.31	-0.37	-0.31	-0.48
	p	0.01	0.006	0.002	0.006	<0.001
Diastolic BP	r	-0.10	-0.14	-0.08	-0.06	-0.21
	p	NS	NS	NS	NS	NS
BMI	r	-0.03	-0.16	-0.10	0.04	-0.16
	p	NS	NS	NS	NS	NS
SWT	r	-0.33	-0.38	-0.44	-0.41	-0.35
	p	0.005	0.001	<0.001	<0.001	0.004
LVEDD	r	0.23	0.28	0.30	0.41	0.41
	p	0.041	0.012	0.017	<0.001	<0.001
LVEDL	r	0.004	0.06	0.06	0.19	0.22
	p	NS	NS	NS	NS	NS

For abbreviations see Tables 1 and 2

On multivariate modeling in FRDA subjects of RV e`and regional LV e`velocities, age, GAA1, GAA2, symptom duration, SWT and LVEDD were added stepwise in that sequence, only significant correlates remained in the model for the next step, and the independent correlations in the final models are shown in Table 7. Because age is a determinant of e`in healthy subjects, and there was colinearity of age with symptom duration, age was included first in all the multivariate models of e`and was an independent predictor in all the models in the absence of symptom duration. The addition of symptom duration in models of e`which included age made no additional contribution to the variances of e`explained and symptom duration is thus not shown in the table. In conjunction with age, GAA1 was an independent predictor of anterior e`(β = -0.25, p = 0.021) and a borderline significant predictor of lateral e`(β = -0.18, p = 0.082), but GAA1 was no longer significant after the addition of SWT to these models. In the final models, GAA1 was only an independent correlate of RV e`, GAA2 was only an independent correlate of septal e`, SWT was an independent predictor of all the e`velocities, whereas LVEDD was only an independent predictor of anterior and lateral e`.

**Table 7:** Multivariate models of RV e`and LV regional e`in individuals with Friedreich ataxia.

		RV e`	Septal e`	Inferior e`	Anterior e`	Lateral e`
Age	β	-0.39	-0.37	-0.41	-0.31	-0.49
	p	<0.001	<0.001	<0.001	0.002	<0.001
GAA1	β	-0.36				
	p	<0.001				
GAA2	β		-0.20			
	p		0.045			
SWT	β	-0.36	-0.47	-0.53	-0.44	-0.41
	p	<0.001	<0.001	<0.001	<0.001	<0.001
LVEDD	β				0.31	0.28
	p				0.001	0.002
	Adjusted r2	0.30	0.30	0.32	0.36	0.42

For abbreviations see Tables 1 and 2

## Discussion

There are a number of new findings about the cardiac effects of FRDA from the present study performed in subjects with a preserved LVEF and no cardiac symptoms. First, there were reductions in RV long-axis systolic and early diastolic velocities and a generalized reduction of regional LV long axis systolic and early diastolic peak velocities compared to control subjects. Second, both RV s`and e`, but none of the regional LV s`or e`velocities, were independently and inversely correlated with GAA1. Third, anterior and lateral s`and septal e`, but none of the other systolic or early diastolic LV velocities, were independently and inversely correlated with GAA2. Fourth, a higher SWT (without indexation for body size) was an independent predictor of lower s`and e`velocities of most LV walls and was also associated with a lower RV e`. Fifth, a smaller LVEDD (also without indexation for body size) was an independent predictor of a lower anterior and lateral e`, but not a predictor of septal or inferior e`or any of the regional LV s`. These findings demonstrate for the first time that there is early impairment of RV long axis function in FRDA, and that the reduction in LV long-axis function in FRDA affects all walls. Despite this apparent uniformity, there were a number of differences between the left and right ventricles, and between the different LV walls, in the associations of TDI velocities with GAA repeats and LV structural change, and thus possibly, in the mechanisms underlying the lower TDI velocities. Furthermore, while some previous studies have reported correlations of GAA2 with cardiac involvement in FRDA, we believe this to be the first study to describe the presence of different and independent associations of GAA1 and GAA2 with measures of cardiac function in FRDA.

There were structural LV changes evident in the FRDA subjects in this study, reflected in LVEDD, SWT, PWT, RWT and LVMI, which are characteristic of the FRDA disease process. The most common abnormality in FRDA is increased RWT, and this is due to the combination of an increase in wall thickness and a reduction in LVEDD [3–5]. LVMI is also increased in FRDA, but LVMI is elevated above the normal range (more than 2 SDs above the normal population mean) on an individual basis less frequently than is RWT [3–5]. The normal sex difference in LVMI was maintained in FRDA, with a higher LVMI evident in males than females in both FRDA and control subjects. There was also a higher LVEDD, SWT and LVEDL in males and the effect of sex on these variables was independent of sex related differences in BSA. There was only minimal evidence of a relationship of GAA repeats with LV structural change. Thus, of SWT, LVEDD, LVMI and LVEDL, only LVEDL was inversely correlated with GAA1 on multivariate analysis, and there were no correlations of LV structural variables with GAA2. Larger studies in FRDA, which have generally included both adults and children, have reported a positive correlation of GAA1 with SWT [3,5] and an inverse correlation with LVEDD [3,21], but have not investigated the relationship of GAA1 with LVEDL.

### Reduced long axis RV function in FRDA

RV function has not been a particular focus of research in FRDA, but there is a previous report that RV regional short-axis contraction can be impaired in FRDA despite preservation of both RV ejection fraction and LV ejection fraction [22]. On the other hand, a more recent study did not find any abnormality of RV long-axis function in FRDA, despite the presence of abnormalities of LV long-axis function [6]. In the present study of adult subjects with FRDA, there was a reduction in both systolic and early diastolic RV TDI long-axis velocities in comparison to control subjects. There are a number of possible reasons why this finding may differ from the other published study on RV long-axis function in FRDA. First, the present study had greater statistical power as there were 78 subjects with FRDA, compared to only 18 subjects in the previous study. Second, the subjects of the present study were all adults, whereas the previous study included a mixture of adults and children. This could be of importance as the effects of FRDA on long-axis function variables could differ between children and adults. Third, the previous study investigated long-axis RV strain, strain rate and early diastolic strain rate and it unclear how these variables relate to TDI systolic and early diastolic velocities, or of the relative sensitivity of strain and TDI techniques to detect abnormal function.

### Reduced long axis LV regional function in FRDA—Relation to LV structural changes

We and others have previously reported that there are abnormalities of LV long-axis systolic and early diastolic motion in FRDA [4,6,7]. The findings of the present study are consistent with these previous studies, but are also an extension of previous findings given that they demonstrate that there are also reductions in anterior and inferior s`and e`in FRDA (as well as septal and lateral s`and e`), and that despite the apparent uniformity of the regional LV abnormalities, there appear to be different determinants of the magnitude of TDI velocities in the different walls. Furthermore, there appears to be patterns in which walls are affected by these determinants, with the contiguous anterior and lateral walls behaving similarly, the septal wall behaving quite differently to the anterior and lateral walls, and the inferior wall being slightly different again. Thus, SWT was an independent determinant of all the LV e`velocities and all the s`velocities except the septal wall. LVEDL was also an independent determinant of all the LV s`velocities except septal s`, whereas LVEDD was an independent determinant of anterior and lateral e`, but not of septal or inferior e`. SWT, LVEDD and LVEDL were chosen as independent variables in the analyses as they represent the characteristic remodeling process of FRDA, which as mentioned above, involves an increase in LV wall thickness and reduction in LV chamber size, both in its short- and long-axes. While it could be postulated that indexation of SWT, LVEDD and LVEDL might be necessary to account for differences in body size, adding BSA to models including the above variables did not improve any of the models of s`or e`(results not shown). The implication therefore is that it is the absolute wall thickness rather than the relative increase in wall thickness above normal for an individual which is the most important determining factor for the long-axis velocities in FRDA.

### Relation of GAA repeats and cardiac structural change with cardiac function

GAA1 has been a consistent predictor of AOS in previous studies in FRDA [1,3,16,23–27], whereas evidence for a correlation of GAA2 with AOS has also been reported but has been less consistent, and the correlation has been less strong [1,3,23,24,27]. A number of studies have also found evidence of associations of both GAA1 and GAA2 with other clinical features of FRDA [1,3,23,24,28]. Some of these studies have reported correlations of both GAA1 and GAA2 with the presence of a cardiomyopathy [1,3,23] and some with the extent of wall thickening [3,28], but it has not generally been investigated whether GAA2 is a predictor of cardiac structure change or dysfunction in FRDA independent of GAA1. Neither has the relationship between GAA1 and GAA2 been investigated in all of the above studies. It is thus important that in the present study, and consistent with one previous report [1], there was a positive correlation between GAA1 and GAA2. This observation provides one possible explanation for a relationship of GAA2 with clinical features of FRDA in previous studies (i.e. collinearity with GAA1) which does not necessarily imply a direct effect of GAA2 on the heart via lower frataxin levels.

In the present study GAA1 was an inverse correlate of AOS, but there was no correlation of GAA2 with AOS. Both RV s`and RV e`were inversely correlated with both GAA1 and GAA2 on univariate analysis, but when included together in a multivariate model, only GAA1 remained as an independent predictor of RV s`and RV e`. On the other hand, GAA2 was a predictor of both anterior and lateral s`and that this was independent of GAA1 indicates that the GAA2 association was not due to any colinearity with GAA1 in this circumstance. Furthermore, that this correlation was also independent of SWT and LVEDD is consistent with there being a GAA2 related functional LV change in FRDA which is independent of the LV structural changes. However, no explanation for this observation is apparent at this time, and a causal relationship cannot be assumed. Thus, the GAA1 association with clinical features in FRDA can be attributed to the established correlation of GAA1 with frataxin levels [29], but a similar relationship of GAA2 with blood or tissue frataxin levels has not been demonstrated. On the other hand, GAA expansion length can differ from one tissue to another [29,30], measurement of cardiac frataxin levels in the early stages of FRDA has not been feasible, and it therefore remains possible that cardiac frataxin levels in the heart may be related to GAA2 and that there may be variation in the levels of frataxin in different parts of the heart.

Analysis of the relationship of TDI velocities with age, AOS and symptomatic duration, and interpretation of the findings, in the present study was necessarily complicated (and potentially confounded) because of the collinearity between these variables and several specific features of the cohort. Indeed, while often not considered, the following issues apply to varying degrees in all cohort studies in FRDA: (1) Age is the sum of AOS and symptom duration, with AOS reflecting both GAA1 and the severity of the underlying disease process [1,29], and symptom duration reflecting in FRDA (as a progressive disease) the time during which deterioration can have occurred following the time of the first presentation with neurological symptoms. Hence, initial consideration of both variables separately could be considered an appropriate technique in FRDA. (2) On the other hand, healthy aging is also associated with impairment of LV long-axis function, particularly e`[15], and so the effect of age on long-axis function merits consideration in its own right. (3) There is the potential for substantial variations in the ratio of AOS to disease duration within individuals in a study population, particularly in adult subjects. (4) Individuals with FRDA die prematurely, with deaths beginning to occur in the teenage years [2] and these individuals cannot be part of a cross-sectional study. Furthermore, exclusion of subjects with reduced LV ejection fraction means that there were additional subjects who could not be included in the current study. These factors must have influenced the makeup of our cohort, e.g. an individual who developed symptoms at age 5 years and either died or developed a reduced ejection fraction at age 29 years can self-evidently never be a 30 year old subject in a cross sectional study of adults with FRDA and a normal ejection fraction. Moreover, it is known that those individuals with the youngest AOS die at the youngest ages [2]. (5) Lastly and of particular importance but rarely mentioned, individuals homozygous for GAA repeats in the *FXN* gene who are yet to develop neurological symptoms, but may already have cardiac changes, cannot currently be part of a cross-sectional cardiac study because there are no screening programs.

There are other limitations of our study. Our cohort was by design limited to adult FRDA subjects with preserved EF and without symptoms or signs of heart failure. Exclusion of individuals without overt cardiac dysfunction will have reduced the variability of TDI velocities, and thus the degree of correlation observed could underestimate the interdependence between some variables. Because of the associated neurologic dysfunction, FRDA subjects are relatively sedentary, and although this was not associated with an increase in BMI in FRDA subjects compared to control subjects in this study, this would likely have resulted in differences in fitness and fat free mass between FRDA and control subjects. A further limitation of the inclusion of sedentary subjects is that it is possible that some subjects were only free of exertional cardiac symptoms because of their lack of ability to exercise.

In conclusion, there are generalized abnormalities of RV and LV regional long axis function in FRDA, but there are also regional differences in the association of the various LV walls long-axis TDI velocities with the small and large alleles of the *FXN* gene and the extent of LV structural change. Further studies are required to confirm these findings and to investigate possible mechanisms which might explain these regional LV and RV differences.

## Supporting information

S1 Data The data for this manuscript is included in the file Long axis function in FRDA & controls July 2018. (XLSX)
